# Characterization of protein isolate from *Sesamum indicum* seed: In vitro protein digestibility, amino acid profile, and some functional properties

**DOI:** 10.1002/fsn3.743

**Published:** 2018-07-27

**Authors:** Temitope O. Fasuan, Saka O. Gbadamosi, Taiwo O. Omobuwajo

**Affiliations:** ^1^ Department of Food Science and Technology Obafemi Awolowo University Ile‐Ife Nigeria

**Keywords:** amino acid profile, functional properties, in vitro protein digestibility, pepsin–pancreatin enzymes, sesame protein isolate, sodium chloride concentration

## Abstract

This work evaluated the impacts of pH and sodium chloride (NaCl) concentration on some functional properties, in vitro protein digestibility, and amino acid profile of sesame (*Sesamum indicum*) protein isolate (SPI) produced using simultaneous recovery of protein and oil technique. The emulsion activity index (EAI), foam capacity (FC), and protein solubility varied with pH and ionic ability. Foam capacity rose with an increase in ionic strength. Protein solubility ranged from 8.39% at pH 4 to 55.08% at pH 10. In vitro protein digestibility of the SPI with pepsin–pancreatin enzyme systems was 89.57%. Amino acid profile showed that essential amino acids constituted 39.48%. The amino acids had good scores well above 50%. The results showed that SPI extracted by aqueous technique could be used as food ingredient, particularly as thickener, binder, and ingredient in baked food products.

## 1 INTRODUCTION

The inadequate availability of protein among human and rapid rise in costs of certain food ingredients sourced from animal have increased interest in plant protein as an alternative for meat protein and dairy products. There is high level of protein deficiency among developing countries in Africa and other continents. The food industry has stimulated research in functional potentials of certain oilseeds (Inyang & Iduh, 1996). Sesame seed, an essential oilseed, is widely cultivated in the tropical and subtropical areas such as Nigeria (Inyang & Iduh, 1996). Sesame seed contains 40%–50% oil and 20%–27% protein (Nzikou et al., 2009). As a result of its composition, it is a potential source of protein. Sesame protein contains adequate amount of essential amino acids such as methionine, cysteine, and tryptophan, which are limiting amino acids in some vegetable protein (like soya). Therefore, sesame protein isolate can be used to enhance the nutritional compositions of certain foods.

In this study, aqueous extraction technique was used, in which protein and oil were simultaneously extracted from *Sesamum indicum* flour without the use of organic solvents such as *n*‐hexane. *N*‐hexane has been associated with potentials of being flammable and explosive and imposes some environmental issues (volatile organic compounds), which are an ongoing concern for the food industry. Aqueous extraction technique is considered a potential alternative to which we can claim as “environmentally clean” process (Tunde‐Akintunde, Oke, & Akintunde, 2012). Aqueous extraction allows the production of food‐grade product. Moreover, reduction in equipment costs and energy consumption is also potentially possible, as oil and protein are recovered simultaneously (Bih‐King & Levente, 2003). Previous works on aqueous extraction process involved the use of enzymes, whereas the application of sodium chloride (NaCl) as surface active agent in collaboration with optimal conditions of pH, temperature, solid‐to‐solvent ratio has not been studied. In the previous parts of this study, oil and protein were simultaneously extracted and the process parameters were optimized (Fasuan, Omobuwajo, & Gbadamosi, 2018). However, in this study, the impacts of pH and ionic power on emulsion activity index and foam capacity, solubility, in vitro protein digestibility, and amino acid profile of the isolated sesame protein through simultaneous recovery of protein and oil (aqueous technique) were discussed.

## 2 MATERIALS AND METHODS

### 2.1 Collection and preparation of material

Sesame seeds were collected from Akure main market, Ondo State, Nigeria. The seeds were cleaned, oven‐dried (SM9053, Uniscope, England) at 50°C, and converted to powder through hammer mill. The powder was sieved using a 630‐micron sieve to obtain product with homogenous particle size.

### 2.2 Preparation of protein isolate

**Figure 1:**
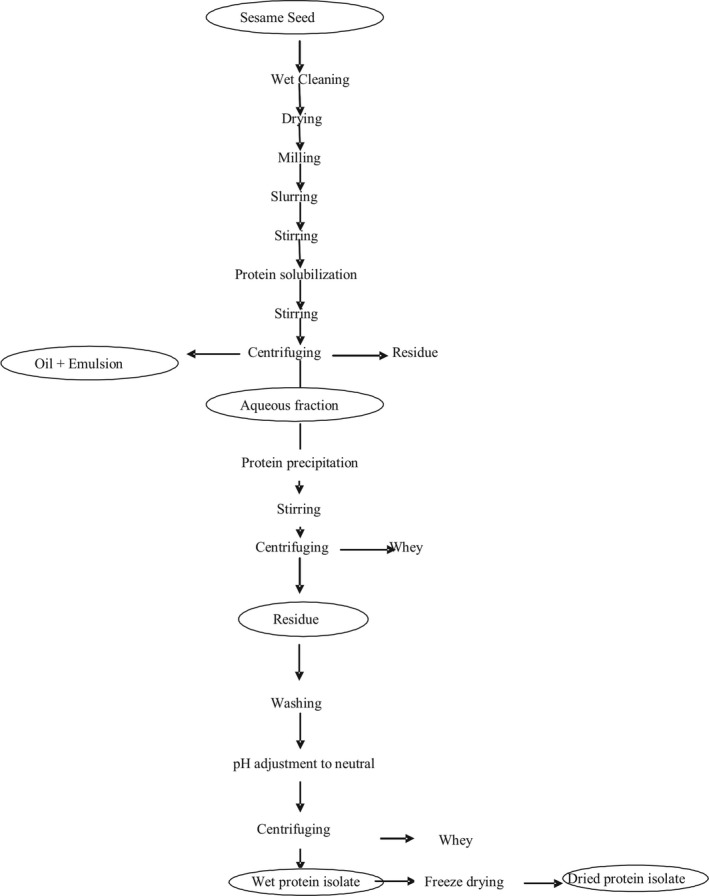
Flowchart for aqueous extraction of protein isolate from sesame seed

Preliminary experiments were conducted to identify the optimal conditions for the independent variables used in this research. The aqueous extraction technique described by Fasuan et al. (2018) and Gbadamosi, Fasuan, and Omobuwajo (2017) was employed. Fifty grams of ground sesame powder was weighed and suspended in 150 ml of 0.1 M NaCl at 47°C and homogenized for 30 min in a thermostat water bath (Julabo, model SW22, Germany). The pH of the mixture was adjusted to 11. The homogenate was then stirred further for 30 min and centrifuged at 3,664 × *g* (0502‐1 Hospibrand, USA) for 20 min at 30°C (Figure 1). The pH of the supernatant was adjusted to 4.5 to separate the soluble protein. The product obtained was homogenized using a magnetic stirrer for 1 hr and centrifuged at 3,664 × *g* (0502‐1 Hospibrand, USA) at 30°C for 15 min. The supernatant was discarded. The residue was washed by dispersing in distilled water, and the pH was adjusted to 7. The resultant product was homogenized using a magnetic stirrer for 30 min and then centrifuged at 3,664 × *g* for 15 min at 30°C. The obtained protein isolate was then freeze‐dried and packaged for further analysis.

### 2.3 Functional properties

#### 2.3.1 Emulsifying activity index and stability

The impact of hydrogen ion concentration and ionic power on emulsifying activity index (EAI) was determined by the turbidimetric method described by Gbadamosi, Abiose, and Aluko (2012). Five hundred milligrams of sesame protein isolate (SPI) was dispersed in 100 ml of distilled water at different NaCl concentrations (0.0, 0.5, and 1.0 M), and the pH was adjusted separately to 2, 4, 6, 8, and 10 with either 1 M HCl or 1 M NaOH. Fifty milliliters of pure edible palm olein oil (Devon King's, PZ Wilmar Foods Ltd, Lagos) was added and homogenized using a blender (O'Qlink, SN2200, China) set at high speed for 60 s. A 50‐μl aliquot of the emulsion was transferred from the bottom of the blender after homogenization and mixed with 5 ml of 0.1% sodium dodecyl sulfate (SDS) solution. The absorbance of the solution obtained was measured at 500 nm using spectrophotometer (722‐2000 Spectronic 20D, England). The absorbance obtained was used to calculate the EAI as shown in Eq. (1).

(1) $$\text{Emulsifying activity index}\;\;(m^{2}/g)\;=\;\frac{2\;\times \;2.303\;\times \;A}{0.25\;\times \;\text{starch weight}\;\;(g)}$$

where 2.303 is turbidimetry constant; *A* is absorbance at 0 min after homogenization; 0.25 is oil volumetric fraction; for a dilute dispersion interfacial, *A* is 2× turbidimetry constant (i.e., 2 × 2.303).

The emulsion stability was determined using Eq. (2).

(2) $$\text{Emulsion stability index}\;=\;\frac{AA\;\times \;\Delta t}{A\;-\;AA}$$

*AA* = absorbance at 10 min, *A* = absorbance at 0 min, ∆*t* = change in time = 10 min.

#### 2.3.2 Foaming capacity and stability

The impact of hydrogen ion concentration and ionic strength on foam capacity and stability was evaluated using the method of Chavan, Mckenzie, and Shahidi (2001) and Ogunwolu, Henshaw, Mock, Santros, and Awonorin (2009). Approximately 500 mg of SPI was dispersed in 100 ml of distilled water at different NaCl concentrations (0.0, 0.5, and 1.0 M); the pH of the protein solution was adjusted separately to pH 2, 4, 6, 8, and 10 with either 1 N HCl or 1 N NaOH and homogenized for 2 min using (O'Qlink, China) blender set at high speed, and then the solution was poured into a 250‐ml measuring cylinder. The percentage ratio of the volume increase to that of the original volume of protein solution in the measuring cylinder was calculated and expressed as foam capacity or whippability (Eq. (3)). Foam stability was expressed as percentage of the volume of foam remaining in the measuring cylinder to that of the original volume after 30 min of quiescent period (Eq. (4)).

(3) $$\text{Foaming capacity}\;(\%)\;=\;\frac{V_{2}\;-\;V_{1}}{V_{1}}\;\times \;100$$

(4) $$\text{Foaming stability}\;(\%)\;=\;\frac{V_{3}\;-\;V_{1}}{V_{1}}\;\times \;100$$

*V*_1_ = volume before whipping (ml), *V*_2_ = volume after whipping (ml), *V*_3_ = volume after standing (ml).

#### 2.3.3 Protein solubility

The effect of pH on protein solubility was determined by a method described by Gbadamosi et al. (2012) with slight modifications. In 250 ml of 0.1 N NaOH, 2.5 g of the sample was dispersed and stirred using a magnetic stirrer (Cenco, Netherlands) for 1 hr, centrifuged (Harrier 15/80 MSE) at 4,500 × *g* for 30 min, and then filtered through Whatman No. 1 filter paper. An aliquot of the filtrate was centrifuged at 12, 000 × *g* for 20 min, and the resulting filtrate obtained was labeled supernatant A. The pH of the remaining filtrate (20 ml) was adjusted separately to pH 2, 4, 6, 8, and 10 with 1 N HCl or NaOH. The solution was then made up to 30 ml and stirred for another 10 min. An aliquot of the mixture was also centrifuged at 12,000 × *g* for 20 min, and the centrifuged solution was labeled as supernatant B. The protein content of supernatants A and B was determined using the modified Lowry method (Markwell, Haas, Biebar, & Tolbert, 1978). One milliliter of supernatants A and B was prepared separately (by adding 50 μl of supernatants A and B separately with 950 μl of distilled water) followed by addition of 3 ml of Lowry's reagent C (mixture of reagents A and B at the ratio 100:1; reagent A: 2% Na_2_CO_3_, 0.4% NaOH, 0.16% sodium chloride, 1% sodium dodecyl sulfate dissolved in distilled water; reagent B: 4% CuSO_4_.5H_2_O dissolved in distilled water) and incubated at ambient temperature for 1 hr. Also, 0.3 ml of distilled Folin–Ciocalteu phenol reagent (1 part of Folin–Ciocalteu phenol with 3 parts of distilled water) was added to the mixture and mixed vigorously using vortex mixer. The tubes were allowed to stand at ambient temperature for 45 min, and the absorbance of the mixture was then measured at 600 nm using spectrophotometer (722‐2000 Spectronic 20 D, England). Exactly 100 μl of bovine serum albumin standard containing 100 μg protein/ml was pipetted, and 900 μl of distilled water was added. The procedure described above was then followed. A plot of absorbance against protein concentration mg/ml gave a straight line curve. The protein concentrations of the sample were extrapolated from the curve.

#### 2.3.4 In vitro protein digestibility

In vitro protein digestibility of the protein isolate was measured using combined methods of Saunder, Connor, Booth, Bickhoff, and Kohler (1973) and as modified by Chavan et al. (2001). Two hundred and fifty milligrams of the sample was suspended in 15 ml of 0.1 M HCl containing 1.5 mg pepsin, followed by gentle shaking for 1 hr at 30 ± 2 °C. The resultant suspension was neutralized with 0.5 M NaOH and treated with 4.0 mg pancreatin in 7.5 ml of phosphate buffer (0.2 M, pH 8.0). The mixture was shaken for 24 hr at 30 ± 2°C. The mixture was then filtered using Whatman No. 1 filter paper, and the residue was washed with distilled water (1:10, w/v), air‐dried, and used for protein determination using Kjeldahl procedure (AOAC, 2000). Protein digestibility was obtained by using Equation (5):

(5) $$\text{In vitro protein digestibility}\;\left(\%\right)\;=\;\left(\frac{I\;-\;F}{I}\right)\;\times \;100$$

where *I* = protein content of sample before digestion, *F *= protein content of sample after digestion.

### 2.4 Amino acid determination

Amino acid profile was determined as described by Blackburn (1978). Amino acid scores were obtained as follows: Total amino acid scores were obtained based on the whole hen's egg amino acid profiles as described by Olaofe, Adeyeye, and Ojugbo (2013). Essential amino acid scores were obtained using the expression (provisional amino acid scoring pattern) as described by Olaofe et al. (2013). The essential amino acid scores were calculated as described by Olaofe et al. (2013) and FAO/WHO (2007). The ratios of total essential amino acids to the total amino acids, total sulfur amino acids, total aromatic amino acids, total neutral amino acids, total acidic amino acids, and total basic amino acids were obtained as described by Olaofe et al. (2013).

## 3 RESULTS AND DISCUSSION

The yield of the protein isolate from *Sesamum indicum* was 18.81 g per 100 g seed flour.

### 3.1 Solubility

**Figure 2:**
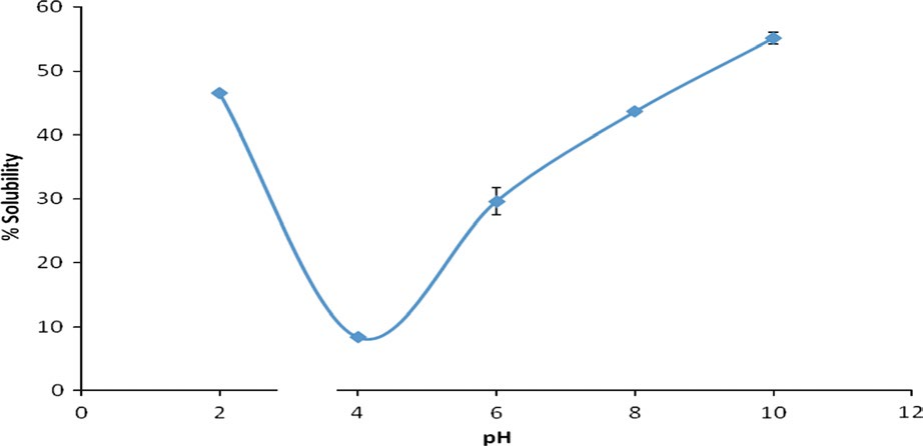
Solubility of sesame protein isolate as a function of pH

The solubility curve for the sesame protein isolate (SPI) is presented in Figure 2. The protein isolate showed least solubility at pH 4 (8.39%), presumably the isoelectric pH, and peak at pH 10 (55.08%). There was a rise in protein solubility below and above the isoelectric pH region. This could be attributed to protein solubility in aqueous solutions, which is a function of pH. At pH values above and below the isoelectric pH, proteins carry a net charge; electrostatic repulsion and ionic hydration promote solubilization of proteins. For most proteins, least solubility takes place at their isoelectric point region, at which electrostatic repulsion and ionic hydration are least (Kanu et al., 2007). The results obtained were in agreement with findings reported by Kanu et al. (2007). The results suggest that the solubility of SPI could be achieved more effectively in alkaline solutions.

### 3.2 Impact of pH and ionic concentration on emulsifying properties

**Figure 3:**
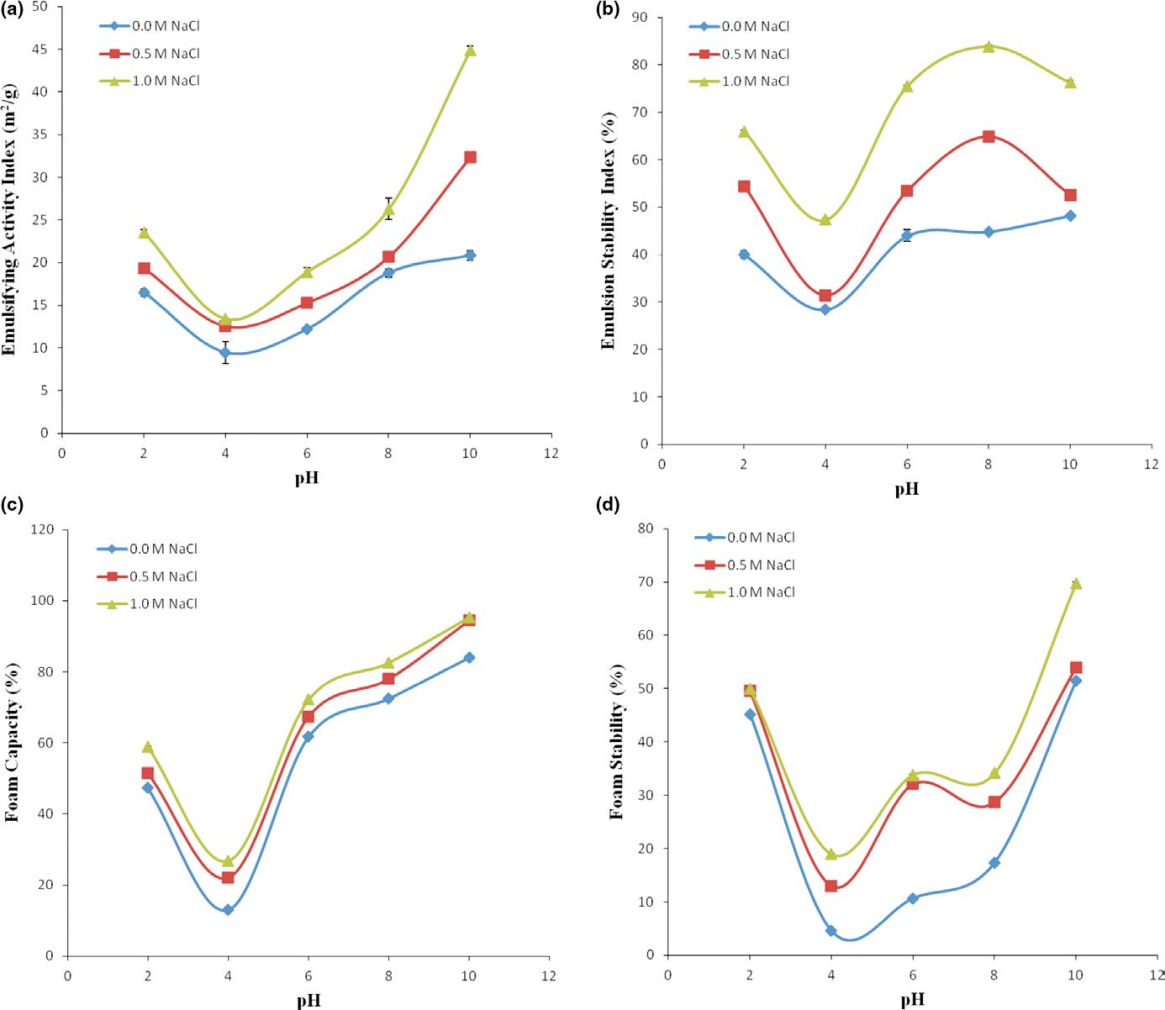
Effects of pH and ionic concentration on emulsifying activity index, emulsion stability index, foam capacity, and foam stability

Figure 3a,b shows the emulsion activity index (EAI) and emulsion stability index (ESI) of the SPI as affected by hydrogen ion concentration and NaCl concentrations (0.5 M and 1.0 M), respectively. The lowest EAI occured at pH 4, which was 9.47 m^2^/g for 0.0 M, 12.57 m^2^/g for 0.5 M, and 13.39 m^2^/g for 1.0 M. There was an increase in EAI at pH < 4 and pH > 4. Alkaline pH enhanced EAI more than acidic pH. This result is similar to the findings of Inyang and Iduh (1996) who stated that protein gets aggregated and destabilized the interfacial membrane when the net charge in the isoelectric region is closed to least. The peak EAI of 20.82 m^2^/g was recorded at pH 10, while the value of 16.49 m^2^/g was recorded at pH 2 for 0.0 M NaCl concentration. Inyang and Iduh (1996) observed EAI of 19.4 m^2^/g at pH 10 and 6.2 m^2^/g at pH 2, for sesame protein concentrate. The observations in this study were similar to the report of Kanu et al. (2007) who reported 21.50 m^2^/g for pH 10 and 17.0 m^2^/g for pH 2. Similar observations were reported for soy protein isolate by Kanu et al. (2007). The study showed that SPI exhibited good EAI at pH 10. This could be associated with the fact that some proteins of plant source can be solubilized at pH below and above the protein isoelectric region.

The EAI of the SPI was equally influenced by the ionic power of the dispersing medium at pH 2–10. The increase in concentration of NaCl from 0.0 M to 1.0 M yielded rise in EAI of the SPI. The rise was from 16.49 m^2^/g and 20.22 m^2^/g for pH 2 and pH 10, respectively, at 0.0 M NaCl concentration to 23.51 m^2^/g and 44.88 m^2^/g for pH 2 and pH 10, respectively, at 1.0 M NaCl concentration. This rise in EAI could be associated with the rise in solubilization of the protein as the ionic power was increased. Emulsifying power is a function of protein surface hydrophobicity.

From Figure 3b, for all NaCl concentrations, the ESI reduced from pH 2 to pH 4 and afterward rose with rising pH. The least ESI occured at pH 4 for all salt concentrations: 28.94%, 31.33%, and 47.37% for 0.0 M, 0.5 M, and 1.0 M NaCl, respectively. These results showed that the addition of NaCl increased the ESI between the pH 4 and pH 8. The emulsion stability of the SPI rose with increased NaCl concentration. The low ESI at low pH and salt concentration was similar to the report of Chavan et al. (2001), which was associated with rise in interactions between the emulsified droplets, as net charge on the protein is reduced by the presence of chloride ions. The EAI is important in processing procedures such as cooking and canning (Ahmed et al., 2012).

### 3.3 Influence of pH and ionic concentration on foaming properties

Figure 3c,d shows the influence of pH and NaCl concentration on foam capacity (FC) and foam stability (FS) of the SPI. The low FC (13%, 22%, and 26.84% for 0.0 M, 0.5 M, and 1.0 M, respectively) occurred at around the isoelectric pH (pH 4). The pH before or beyond 4 caused rise in FC for all NaCl concentrations. The peak (66.50% for 0.0 M, 94.50% for 0.5 M, and 95.42% for 1.0 M) occurred at pH 10. The low FC at pH 4 could be associated with low solubility at this pH. The higher FC at pH 10 could be associated with the rise in net charges on the protein, which reduced the hydrophobic interactions but enhanced the pliability of the protein (Fligner & Mangino, 1991). The result obtained was consistent with the report of Kanu et al. (2007) for sesame protein isolate. The foam stability reduced with rise in pH from pH 2 to pH 4. The peak foam occurred at pH 10. The results indicated that rise in NaCl concentration from 0.0 M to 1.0 M increased the foam stability. From Figure 3c,d, the foam capacity rose with rise in NaCl concentration. For pH 2, the rise was from 47.34%, when suspended in distilled water (0.0 M concentration), to 59% at 1.0 M concentration. For pH 4 (isoelectric pH), the rise was from 13% at 0.0 M to 26.84% at 1.0 M. For pH 10, the rise was from 84% at 0.0 M to 95.42% at 1.0 M. This rise is consistent with results reported by Kanu et al. (2007) for sesame protein isolate and soy protein isolate, and by Inyang and Iduh (1996) for sesame protein concentrate. The rise could be associated with rise in solubilization of protein as ionic power rose. The SPI may find application in foods that require good foaming property such as salad dressing.

### 3.4 In vitro protein digestibility

*In vitro* protein digestibility of sesame protein isolate using pepsin–pancreatin enzyme systems showed that the SPI had digestibility of 89.57%. This result was higher than the value of 30.50% reported by Maha, Abdullahi, Isam, and Elfadil (2009) for uncooked soybean flour and 35% reported by Gbadamosi et al. (2012) for conophor protein concentrate, but similar to the value of 90% reported by Wanasundara and Shahidi (1997) for flaxseed protein isolate. The high digestibility of the SPI could be attributed to fall in the nonprotein compounds, particularly polysaccharides, as well as rise in the availability of the proteins for enzymatic activities (Adiamo, Gbadamosi, & Abiose, 2016).

### 3.5 Amino acid profile

Table 1 shows the amino acid (AA) profile for the SPI. Glutamic acid had the highest concentration (16.54 g/100 g) followed by aspartic acid (9.88 g/100 g), and they are acidic amino acids (AAA). Table 1 shows that the limiting amino acids were sulfur amino acids (methionine 1.87 g/100 g; cysteine 0.15 g/100 g). The highest essential amino acid (EAA) was leucine (7.57 g/100 g). All the values obtained for AA of the sample were similar to previous work on sesame protein isolate. The value of leucine (7.57 g/100 g), isoleucine (4.85 g/100 g), methionine (1.87 g/100 g), arginine (7.45 g/100 g), serine (6.62 g/100 g), valine (5.44 g/100 g), and histidine (2.25 g/100 g) were similar to the report of Fleddermann et al. (2013) for sesame protein isolate: leucine (7.68 g/100 g), isoleucine (4.04 g/100 g), methionine (1.17 g/100 g), arginine (7.36 g/100 g), serine (5.36 g/100 g), valine (4.16 g/100 g), and histidine (2.44 g/100 g). The values of lysine (5.06 g/100 g), aspartic acid (9.88 g/100 g), proline (4.08 g/100 g), alanine (2.83 g/100 g), and tryptophan (1.25 g/100 g) were similar to the report of Cervantes‐Pahm and Stein (2010) for sesame protein isolate: lysine (4.78 g/100 g), aspartic acid (8.49 g/100 g), proline (3.70 g/100 g), alanine (3.13 g/100 g), and tryptophan (1.03 g/100 g). The essential amino acids of the SPI near those of reference protein (hen's egg) except that of methionine (Table 1). All the amino acids have good scores except methionine that was 58%. The AA scores ranged from 58% to 138%.

**Table 1:** Amino acid profile and amino acid scores of simultaneously recovered sesame protein isolate with respect to whole hen's egg

Amino acid	Sesame protein isolate (g/100 g)	Hen's egg^a^ (g/100 g)	Soy protein^b^ (g/100 g)	Pea protein^c^ (g/100 g)	Amino acid score
Glycine	2.06	3.00	4.18	4.32	0.69
Alanine	2.83	5.40	4.26	4.27	0.52
Serine	6.62	7.90	5.49	4.79	0.84
Proline	4.08	3.80	5.49	3.76	1.07
Valine	5.44	7.50	4.80	3.89	0.73
Threonine	4.85	5.10	3.86	3.59	0.95
Isoleucine	4.85	5.60	4.54	3.33	0.87
Leucine	7.57	8.30	7.78	6.58	0.91
Aspartic acid	9.88	10.70	11.70	10.68	0.92
Lysine	5.06	6.20	6.38	6.84	0.82
Glutamic acid	16.54	12.00	18.70	16.92	1.38
Methionine	1.87	3.20	1.26	1.03	0.58
Phenylalanine	6.34	5.10	4.94	4.19	1.24
Histidine	2.25	2.40	2.53	2.52	0.94
Arginine	7.45	6.10	7.23	6.84	1.22
Tyrosine	6.61	4.00	3.14	3.16	1.65
Cysteine	0.15	1.80	1.33	1.55	0.08
Tryptophan	1.25	—	1.28	0.94	—

a Olaofe et al. (2013).

b FAO (1992).

c Leterme, Monmart, and Baudart (1990).

Most of essential amino acids of the SPI were found superior to those of pea and soy proteins. These include valine, threonine, isoleucine, methionine, phenylalanine (Table 1); leucine and tryptophan of the SPI showed superiority over pea protein. However, SPI is deficient in lysine and histidine relative to pea and soy proteins (Table 1).

The AA distribution in the sesame protein isolate is shown in Table 2. The ratios of total essential amino acid (TEAA) to total amino acid (TAA) in the sample (39.48% with histidine, 35.23% without histidine) near 39% stated to be sufficient for ideal protein food for infants, well above 26% for children and 11% for adults (FAO/WHO, 1990; Olaofe et al., 2013). Arginine is essential for children. The value of arginine (7.45 g/100 g) was above the reference protein (6.10 g/100 g).

**Table 2:** Amino acid distribution in sesame protein isolate (dry weight)

Parameter	Quantity (g/100 g)
Total amino acid (TAA)	95.70
Total nonessential amino acid (TNEAA)	56.22
Total essential amino acid (TEAA)	
With histidine	39.48
Without histidine	35.23
Total neutral amino acid (TNAA)	54.52
Total acidic amino acid (TAAA)	26.42
Total basic amino acid (TBAA)	14.76
Total sulfur amino acid (TSAA)	2.02
Total aromatic amino acid (TArAA)	14.20
% TNEAA	58.75
%TEAA	
With histidine	41.25
Without histidine	36.81
% TNAA	56.97
% TAAA	27.61
% TBAA	15.42
% TSAA	2.11
% Cystine TSAA	7.11
% TArAA	14.84

The AA scores of the sesame protein isolate with respect to the reference protein are shown in Table 3. The AA scores with respect to provisional AA scoring pattern of the FAO for essential amino acids were well above 100% except sulfur‐containing AA (methionine + cystine) and lysine that were 58% and 92%, respectively (Table 3). The amino acid scores with regard to the requirements of infants, preschool children, adolescents, and adults are shown in Table 4. All the scores were well above 100% except those of sulfur‐containing AA (methionine + cystine) and lysine that were 81% and 87%, respectively.

**Table 3:** Amino acid scores with respect to provisional amino acid scoring pattern of the FAO (amino acid values in g/100 g)

Amino acid	Sesame protein isolate	Scoring values^a^	Amino acid scores
Isoleucine	4.85	4.00	1.21
Leucine	7.57	7.00	1.08
Lysine	5.06	5.50	0.92
Met + Cys (TAAA)	2.02	3.50	0.58
Phe + Tyr	12.95	6.00	2.16
Threonine	4.85	4.00	1.21
Tryptophan	1.25	1.00	1.25
Valine	5.44	5.00	1.09

a Olaofe et al. (2013).

**Table 4:** Amino acid scores with respect to requirements (amino acid values in g/100 g)

Amino acid	Sesame protein isolate	Infant^a^	Preschool children^a^	Adolescent^a^	Adult^a^
Leucine	7.57	7.30	5.40	4.40	3.90
Isoleucine	4.85	3.60	2.70	2.20	2.00
Lysine	5.06	6.40	4.50	3.50	3.00
Met + Cys	2.02	3.10	2.20	1.70	1.50
Phe + Tyr	12.95	5.90	4.00	3.00	2.50
Tryptophan	1.25	0.95	0.64	0.48	0.40
Threonine	4.85	3.40	2.30	1.80	1.50
Valine	5.44	4.90	3.60	2.90	2.60
Histidine	2.25	2.20	1.50	1.20	1.00

a FAO/WHO (2007).

The results showed that the amino acid profile of SPI extracted by aqueous technique could be used for food enrichment and as food supplement.

## 4 CONCLUSION

Emulsion activity index, foam capacity, and solubility of sesame protein isolate varied with pH and NaCl concentration. Rise in ionic strength of the dispersing medium yielded increase in these properties. The essential amino acids of the sesame protein isolate (SPI) had similar score to those of reference protein (hen's egg) in quality. The total amino acid was 95.70 g/100 g SPI. The high value of the in vitro protein digestibility showed that SPI could be used to enrich and as supplement in some food systems, especially in developing countries where protein deficiency is a major health challenge for children. The results of ESI and FC suggest that SPI could be used as functional food ingredient.

## CONFLICTS OF INTEREST

The authors state that there were no conflicts of interest.
